# Cumulative physical workload and mobility limitations in middle-aged men and women: a population-based study with retrospective assessment of workload

**DOI:** 10.1007/s00420-019-01399-3

**Published:** 2019-01-18

**Authors:** Anne Møller, Minna Mänty, Lars L. Andersen, Volkert Siersma, Rikke Lund, Ole Steen Mortensen

**Affiliations:** 1 Department of Occupational and Social Medicine, Copenhagen University Hospital Holbaek, Smedelundsgade 60, 4300 Holbaek, Denmark; 2 The Research Unit for General Practice and Section of General Practice, Department of Public Health, University of Copenhagen, Copenhagen K, Denmark; 3 Department of Public Health, Faculty of Medicine, University of Helsinki, Helsinki, Finland; 4 Department of Research, Development and Innovation, Laurea University of Applied Sciences, Vantaa, Finland; 5 National Research Centre for the Working Environment, Copenhagen, Denmark; 6 Section of Social Medicine, Department of Public Health, University of Copenhagen, Copenhagen, Denmark; 7 Center for Healthy Aging, University of Copenhagen, Copenhagen, Denmark; 8 Physical Activity and Human Performance group, SMI, Department of Health Science and Technology, Aalborg University, Aalborg, Denmark

**Keywords:** Physical workload, Cumulative exposure, Job exposure matrix, Mobility limitations

## Abstract

**Purpose:**

To assess the association between exposure to physical workload throughout working life and risk of mobility limitations in midlife in a population-based Danish cohort.

**Methods:**

The study was cross-sectional with a retrospective exposure assessment, and data were from a questionnaire used in the Copenhagen Aging and Biobank. Cumulative physical workload was estimated by combining information about the participants’ employments and data from a job exposure matrix. Daily amount of lifting was standardised in ton-years (lifting 1000 kg/day/year) and grouped in 5 exposure groups (no/minor (1–2 ton-years)/low (3–10 ton-years)/moderate (11–20 ton-years)/high exposure (> 20 ton-years)). The outcome was self-reports of mobility limitations (running 100 m, walking 400 m, and climbing stairs to the 2nd floor) in midlife. The association between exposure and outcome was analysed using logistic regression models.

**Results:**

We included 4996 men and 2247 women, mean age 56 years. 21% of men and 10% of women were in the highest exposure-group (> 20 ton-years). Higher cumulative exposure was associated with higher odds for mobility limitations. Exposure to more than 20 ton-years compared to no exposure increased the odds for limitations in walking, age-adjusted odds ratio (OR) 3.2 (95% CI: 2.4–4.3) for men, 2.3 (1.4–3.8) for women. Corresponding results for running: 2.5 (2.2–3.0) for men, 1.6 (1.2–2.2) for women, and for limitations in climbing stairs: 4.2 (3.3–5.2) for men, 1.7 (1.2–2.4) for women. Results were attenuated when confounders were added.

**Conclusions:**

Exposure to physical workload throughout working life is associated with higher odds for mobility limitations in midlife.

## Background

“The health benefits of physical activity and exercise are clear; virtually everyone can benefit from becoming more physically active” (Warburton and Bredin 2017) However, research has revealed opposing effects of physical activity at work and in leisure time according to outcomes such as sickness absence, cardiovascular disease and death (Holtermann et al. 2011; Coenen et al. 2018). According to outcomes related to physical performance, physical strength and mobility, the evidence also suggests opposing effects of physical activity at work and in leisure time. A few older studies have indicated a positive effect of physical activity on physical performance, but studies also suggest different impact on different parts of the body (Torgén et al. 1999; Schibye et al. 2001; Møller et al. 2013). However, most studies indicate a negative effect of physical workload on later physical performance, which could be explained by insufficient recovery (de Croon et al. 2003; Mohren et al. 2010), though the causal relationship is not fully understood yet. The deterioration of physical performance by physical workload could also be explained by theories from life course epidemiology about the effect of cumulative exposures on later health (Kuh et al. 2003). Knowledge about the effect of physical workload could be used to prevent work-related deterioration of physical performance.

Mobility is defined as the ability to move independently from one location to another (Vasunilashorn et al. 2009) and is considered a clinically relevant outcome measure in epidemiology since mobility limitations influence daily life, including involvement in social activities and labour market participation. Furthermore, limitations in mobility are prognostic for subsequent physical disability (Guralnik et al. 1995), dependency on others (Perera et al. 2016), and mortality (Studenski et al. 2011; Bergland et al. 2017). However, among studies of the association between physical workload and later health, few have used mobility limitations as an outcome measure. In a prospective study, Hinrichs et al. found an inverse relationship between occupational and leisure-time physical activity on risk of mobility limitations in old age(Hinrichs et al. 2014). They performed a prospective follow-up study among public-sector workers in Finland. Baseline information about occupational physical workload was obtained in 1981, and the incidence of mobility limitations was studied during 28 years of follow-up. The results suggest harmful effects of heavy physical workload on later mobility. Leino-Arjas et al. (Leino-Arjas et al. 2004) performed a follow-up study over 28 years in a cohort of metal industry employees (average age 63 at the end of follow-up). Physical workload was assessed at three stages: at baseline, after 10, and 28 years. On the other hand, the outcome and mobility limitations were only assessed after 28 years. The results indicated that a high level of physical workload at baseline increased later risk of mobility limitations. In addition, a recent study from our research group showed that high physical workload assessed at baseline was associated with higher risk of mobility limitations during a 6-year follow-up, whereas leisure-time physical activity was associated with a lower risk of mobility limitations (Mänty et al. 2014). However, neither of these studies (Leino-Arjas et al. 2004; Mänty et al. 2014; Hinrichs et al. 2014) included a cumulative measure of physical workload. The aim of the present study is therefore to include a cumulative assessment of physical workload throughout working life.

In the present study, we use data from the Copenhagen Aging and Midlife Biobank (CAMB), a cohort that we have previously used to investigate how cumulative physical workload influences objectively measured physical performance in midlife (grip strength and chair-rise performance)(Møller et al. 2013, 2015).

The aim of the present study is to assess the association between cumulative physical workload and self-reported mobility limitations in midlife. Unlike previous studies in this field, we have access to a validated Job Exposure Matrix (Rubak et al. 2014), through which we can calculate participants’ cumulative physical workload throughout working life.

## Methods

This population-based study with retrospective assessment of workload is part of the Copenhagen Aging and Midlife Biobank (CAMB) (Avlund et al. 2014; Lund et al. 2015) established in 2009. The data collection in CAMB was made between April 2009 and March 2011 and included a postal questionnaire and an invitation to a health examination at the National Research Centre for the Working Environment (NRCWE) in Copenhagen, Denmark (Lund et al. 2015). CAMB is based on three existing Danish cohorts: The Metropolit 1953 Danish Male Birth Cohort (MP) including men born in 1953, The Danish Longitudinal Study on Work, Unemployment and Health (DALWUH) with participants born 1949 or 1950 and the Copenhagen Perinatal Cohort (CPC) including participants born between 1959 and 1961(Lund et al. 2015). In total, 17,937 cohort members were invited to participate in the CAMB cohort, 7750 from MP, 4906 from DALWUH, and 5282 from CPC. Of these, 40% answered the questionnaire (respondents): 4993 men (of whom 63% were from MP, 18% from DALWUH, and 19% from CPC), and 2247 women (of whom 47% were from DALWUH and 53% from CPC. The MP cohort did not include women). 31% (5576 participants) attended the health examination [see (Lund et al. 2015) for further information]. Compared to non-respondents, respondents were better educated, and more respondents were employed, though all social strata were represented in the cohort (Lund et al. 2015). The study protocol was approved by the local ethics committee (number H-A-2008-126) and the Danish Data Protection Agency (number 2008-41-2938).

### Exposure

The retrospective assessment of cumulative exposure to physical workload was based on information about job history from the CAMB questionnaire combined with data from a job-exposure matrix. The CAMB questionnaire provided job titles and length of service for the participants’ five longest held occupations. Each participant’s job history was coded according to the 1988 revision of the Danish version of the International Standard Classification of Occupations register (D-ISCO 88) (Møller et al. 2012). Information about physical workload in Danish jobs (linked to D-ISCO-88 codes) was retrieved from a Danish job exposure matrix; the Lower Body Job Exposure Matrix (Lower Body JEM) (Rubak et al. 2014). The Lower Body JEM was established to study associations between exposures at work and the risk of osteoarthritis in the lower limbs. The Lower Body JEM contains information about daily amount of lifting, standing/walking and kneeling in 121 job groups with homogeneous exposure patterns. The assessment of exposure was made by five occupational health physicians who rated the mean number of hours per day spent standing/walking, kneeling/squatting, and exposure to whole-body vibration (in half-hour intervals). Sitting was also assessed, so that the experts could ensure that exposures added up a full working day defined as 8 hours. According to lifting, the experts were asked to estimate the total load lifted (kg/day) and the frequency of lifting loads weighing > 20 kg (times/day). The ratings were compared and gross outliers were discussed at a panel meeting, and the ratings were tested for face-validity in two other experts in the field. (Rubak et al. 2014). In this study, the primary exposure of interest is “cumulative physical workload”, and we use information about lifting as a proxy measure of physical workload.

The cumulative physical workload for a study participant was expressed as the number of years incurred by a standard daily exposure. Thus, the years of employment in each job (retrieved from the questionnaire) were multiplied by the corresponding daily amount of lifting (in kilograms) retrieved from the Lower Body JEM, and then calculated for the participants’ entire working life. In this way, workload was standardized as ton-years (lifting 1000 kg each working day in 1 year).

From our previous work (Møller et al. 2013), we know that the distribution of workload is skewed. Many participants have no physical workload during working life, a large group has moderate workload, and some participants have a high level of cumulative physical workload throughout working life. In this study, the cumulative physical workload is divided into five groups: (1) no exposure (reference group), (2) minor exposure (> 0–2 ton-years), (3) low exposure (> 2–10 ton-years), (4) moderate exposure (> 10–20 ton-years), and (5) high exposure (> 20 ton-years).

### Outcome

Mobility limitations were assessed by questions about participants’ ability to walk 400 meters, run 100 meters, and climb stairs to the second floor. The first question was: “Can you usually walk 400 m (0.25 miles) without resting?” with the response categories: “Easily”, “With little difficulty”, “With much difficulty”, “Not at all”. (Question was retrieved from The Danish Health Interview Survey, 1987 (SUSY), The National Institute of Public Health, University of Southern Denmark). We categorised the answers in: (1) no limitations (“Easily”) or (2) limitations (“with little” or “with much difficulty”, or not able to walk 400 meters at all). The second and third questions were: “Does your health now limit you in the following two activities: (a) Running 100 meters, (b) Climbing stairs to the 2nd floor?” with response categories: “Yes, limits a lot”, “Yes, limits a little”, “No, does not limit at all”. We dichotomized the answers to: (1) no limitations (“does not limit at all”) and (2) limitations (“limits a little”/“a lot”). These questions are modified from SF-36 Health Survey (Bjorner et al. 1998).

### Confounders and mediators

The associations between cumulative physical workload and mobility limitations can be influenced by various possible confounders and mediators. Age is a confounder and included in our analyses; however, the age span of the cohort is narrow due to the original cohorts. Chronic diseases and pain could be confounders as well as mediators. Chronic disease and pain could be caused by exposures and influence mobility. However, chronic diseases and pain could also be a confounder and influence the work ability and thereby the exposure and the outcome (mobility). The CAMB questionnaire provided information about the number of chronic diseases among participants, and these were grouped in three categories: “no”, “one”, and “two or more” chronic diseases. The diseases counted were asthma, diabetes, hypertension, angina pectoris, stroke, myocardial infarction, bronchitis, emphysema, rheumatoid arthritis, osteoarthritis, cancer, anxiety, depression/other psychiatric diseases and back pain. Unfortunately, we had no information about severity of diseases. Pain in nine regions of the body was summarized (neck, shoulders, upper part of back, elbows, lumbar region, hands/wrists, hips, knees and ankles); the minimum score was 9 (no pain in any of the regions) and the maximum was 81 (worst possible pain in all 9 regions). In the questionnaire, we have information about lifestyle factors which can be confounders or mediators in the association between physical workload and mobility limitation. High physical workload can lead to less energy or motivation for leisure-time physical activity (LTPA) which is known to influence mobility. However, LTPA could also be a confounder influencing the capacity of physical work and mobility. LTPA was categorised as “active” (≥ 4 h a week), “moderately active” (< 4 h a week) and “sedentary” (0 h). Smoking and alcohol consumption was seen as confounders. Smoking history was calculated as pack-years (defined as 20 cigarettes or an equal amount of tobacco smoked each day for 1 year), and current alcohol consumption was categorized in units of alcohol per week.

### Statistical analysis

The effect of physical workload was assumed to be gender-specific, and all analyses were performed separately for each sex, as suggested by Silverstein et al. (Silverstein et al. 2009). We present numbers, frequencies, mean values and standard deviations of the characteristics of the study population. Associations between ton-years and self-reported mobility limitations were assessed as odds ratios (OR) with corresponding 95% confidence intervals (95% CI) from logistic regression models. Those reporting any limitations were recorded as having the outcome, and responders without limitations were used as a reference group. Initial models were adjusted for age (Model 1). Subsequent models were additionally adjusted for chronic diseases and pain (Model 2), variables that can be both confounders or mediators as mentioned above, and finally, lifestyle factors: LTPA, smoking and alcohol use (Model 3). Observations with missing values for specific covariates were omitted from analyses that included these covariates [*n* = 4996 in model 1 and *n* = 4514 in model 3 (men) and *n* = 2247 in model 1 and *n* = 1993 in model 3 (women)]. All analyses were performed in PROC LOGISTIC (SAS 9.4). The level of statistical significance was 5%.

The associations between objective measures and mobility limitations were studied in PROC FREQ (SAS 9.4) using gamma coefficients.

## Results

Mean age for men was 57.6 years (range 53–59 years), and for women 55.0 (range 52–63 years). Mean duration of working life was 30 years for men and 26 years for women based on summation of the five longest held employments listed in the questionnaire. Descriptive data are presented in Table 1. More than a third of the participants had two or more chronic diseases. In general, about one in ten had limitations walking 400 m, one in five had limitations climbing stairs, and one in three had limitations running 100 meters.

**Table 1:** Characteristics of the study population, exposures and outcome

Variable	Men		Women	
	*N*		*N*	
Age (years), mean (SD)	4996	57.6 (3.5)	2247	55.0 (4.9)
Duration of working life (years), mean (SD)	4800	30.3 (8.2)	2165	26.4 (8.6)
Smoking, pack-years, mean (SD)	4754	19.5 (25.5)	2109	11.9 (15.3)
Alcohol consumption, units/week, mean (SD)	4864	14.6 (14.4)	2169	7.1 (7.3)
Pain index^a^	4937	19.7 (10.7)	2233	23.7 (13.1)
Chronic diseases^b^, *n* (%)	4942		2231	
No disease		1587 (32.1)		721 (32.3)
One disease		1657 (33.5)		708 (31.7)
Two or more diseases		1698 (34.3)		802 (36.0)
Leisure-time physical activity^c^, *n* (%)	4904		2210	
Medium/hard		1622 (33.1)		567 (25.7)
Light		2698 (55.0)		1453 (65.7)
Sedentary		584 (11.9)		190 (8.6)
Ton-years^d^, *n* (%)	4800		2165	
0 ton-years		2033 (42.4)		1152 (53.2)
>0–2 ton-years		285 (5.9)		188 (8.7)
>2–10 ton-years		873 (18.2)		379 (17.5)
>10–20 ton-years		625 (13.0)		228 (10.5)
>20 ton-years		984 (20.5)		218 (10.1)
Walk 400 m without resting^e^, *n* (%)	4848		2247	
No limitations		4501 (90.1)		2019 (89.8)
Limitations		495 (9.9)		228 (10.2)
Climbing stairs to second floor^f^, *n* (%)	4996		2247	
No limitations		4142 (82.9)		1769 (78.7)
Limitations		854 (17.1)		478 (21.3)
Running 100 m^f^, *n* (%)	4996		2247	
No limitations		3545 (71.0)		1501 (66.8)
Limitations		1451 (29.0)		746 (33.2)

*SD* standard deviation

^a^Summation of pain in nine regions of the body. Minimum score is nine (no pain in any of the regions) and maximum is 81 (worst possible pain in all nine regions)

^b^Asthma, diabetes, hypertension, angina, stroke, bronchitis, chronic obstructive pulmonary disease, rheumatoid arthritis, osteoarthritis, cancer, anxiety, depression, psychiatric diseases, and back disease

^c^Medium/ hard: > 4 h a week, light: < 4 h a week, sedentary: reading/watching television in leisure-time

^d^Amount of lifting during working life. One ton-year is lifting 1000 kg each day in one year

^e^No limitations (“easily”) or limitations (“with little” or “with much difficulty”, or “not able to walk 400 m at all”)

^f^No limitations (health does not limit at all) and limitations (health limits a little/a lot)

**Fig. 1:**
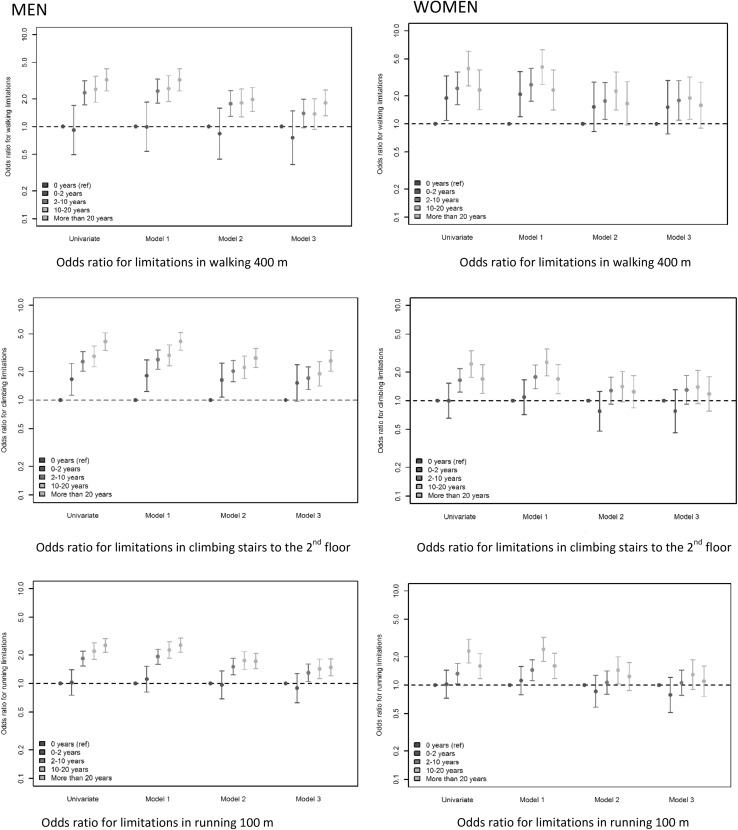
Odds ratio for mobility limitations in men and women

In unadjusted analyses, cumulative physical workload was associated with risk of limitations in mobility (Table 2 gives the exact numbers and the associations are visualised in Fig. 1).

**Table 2:** OR for mobility limitations according to exposure to cumulative years of lifting through working life measured as ton-years (CI: confidence interval)

	Men	Unadjusted (*n* = 4996)			Model 1 (*n* = 4800)			Model 2 (*n* = 4786)			Model 3 (*n* = 4514)		
	*N*	OR	95% CI		OR	95% CI			95% CI			95% CI	
Walking													
0 ton-years	2033	1 (ref)			1 (ref)			1 (ref)			1 (ref)		
> 0–2 ton-years	285	0.917	0.496	1.695	0.995	0.537	1.842	0.841	0.444	1.592	0.759	0.388	1.484
> 2–10 ton-years	873	2.338	1.727	3.166	2.434	1.796	3.300	1.779	1.289	2.454	1.391	0.974	1.985
> 10–20 ton-years	625	2.547	1.837	3.530	2.595	1.870	3.600	1.813	1.281	2.565	1.373	0.935	2.015
> 20 ton-years	984	3.232	2.449	4.265	3.228	2.444	4.262	1.977	1.468	2.662	1.807	1.311	2.491
Running													
0 ton-years	2033	1 (ref)			1 (ref)			1 (ref)			1 (ref)		
> 0–2 ton-years	285	1.027	0.753	1.401	1.115	0.816	1.524	0.967	0.691	1.353	0.898	0.630	1.281
> 2–10 ton-years	873	1.839	1.536	2.203	1.921	1.601	2.305	1.506	1.234	1.837	1.300	1.050	1.609
> 10–20 ton-years	625	2.200	1.806	2.681	2.257	1.850	2.755	1.752	1.409	2.180	1.430	1.131	1.810
> 20 ton-years	984	2.530	2.136	2.995	2.544	2.146	3.015	1.724	1.429	2.081	1.484	1.213	1.816
Climbing stairs													
0 ton-years	2033	1 (ref)			1 (ref)			1 (ref)			1 (ref)		
> 0–2 ton-years	285	1.659	1.129	2.436	1.810	1.229	2.664	1.625	1.075	2.456	1.516	0.976	2.353
> 2–10 ton-years	873	2.555	2.020	3.231	2.670	2.108	3.381	2.022	1.568	2.608	1.702	1.291	2.245
> 10–20 ton-years	625	2.897	2.250	3.729	2.966	2.301	3.822	2.210	1.681	2.905	1.899	1.414	2.551
> 20 ton-years	984	4.141	3.345	5.128	4.163	3.359	5.159	2.775	2.199	3.502	2.596	2.024	3.332

	Women	Unadjusted (*n* = 2247)			Model 1 (*n* = 2165)			Model 2 (*n* = 2165)			Model 3 (*n* = 1993)		
	*N*	OR	95% CI		OR	95% CI			95% CI			95% CI	
Walking													
0 ton-years	1152	1 (ref)			1 (ref)			1 (ref)			1 (ref)		
> 0–2 ton-years	188	1.894	1.093	3.282	2.088	1.199	3.635	1.526	0.829	2.808	1.514	0.781	2.937
> 2–10 ton-years	379	2.410	1.609	3.610	2.628	1.746	3.955	1.764	1.122	2.772	1.791	1.098	2.920
> 10–20 ton-years	228	3.922	2.564	6.000	4.077	2.656	6.257	2.253	1.407	3.610	1.895	1.122	3.201
> 20 ton-years	218	2.317	1.419	3.781	2.313	1.414	3.785	1.659	0.970	2.838	1.591	0.905	2.796
Running													
0 ton-years	1152	1 (ref)			1 (ref)			1 (ref)			1 (ref)		
> 0–2 ton-years	188	1.021	0.724	1.438	1.120	0.791	1.584	0.861	0.583	1.270	0.787	0.511	1.212
> 2–10 ton-years	379	1.326	1.034	1.701	1.443	1.121	1.858	1.063	0.800	1.413	1.057	0.776	1.438
> 10–20 ton-years	228	2.299	1.720	3.074	2.403	1.791	3.226	1.438	1.033	2.001	1.292	0.897	1.859
> 20 ton-years	218	1.599	1.182	2.163	1.604	1.181	2.179	1.239	0.880	1.743	1.103	0.762	1.596
Climbing stairs													
0 ton-years	1152	1 (ref)			1 (ref)			1 (ref)			1 (ref)		
> 0–2 ton-years	188	0.999	0.656	1.522	1.090	0.713	1.666	0.773	0.479	1.248	0.776	0.462	1.302
> 2–10 ton-years	379	1.638	1.233	2.177	1.777	1.332	2.371	1.277	0.921	1.769	1.301	0.916	1.847
> 10–20 ton-years	228	2.428	1.762	3.346	2.522	1.824	3.487	1.407	0.974	2.033	1.392	0.933	2.079
> 20 ton-years	218	1.690	1.194	2.392	1.691	1.191	2.400	1.241	0.839	1.836	1.180	0.779	1.787

Walking: limitations in walking 400 m without resting. Dichotomized to “no difficulty” or “little or much difficulty or not able at all”. No difficulty is used as reference group

Running: limitations in running 100 m. Dichotomized “not limited at all” and “limited a little/a lot”. Not limited is used as reference group

Climbing stairs: limitations in climbing stairs to the second floor. Dichotomized to “not limited at all” and “limited a little/a lot”. Not limited is used as reference group

Ton-years: amount of lifting during working life. One ton year is lifting 1000 kg each day in one year

Model 1: including age. Model 2: Model 1 and chronic diseases and pain. Model 3: Model 2 and life style factors

### Limitations in walking 400 m

In all models, men had higher odds of mobility limitations with higher level of physical workload. Among women, more exposure-years were associated with higher odds of mobility limitations except from women with high exposure. Women with high exposure had lower odds of mobility limitations compared to women with low or moderate exposure. Introduction of confounders and mediators in the analysis attenuated the associations for both men and women (Models 2 and 3).

### Limitations in running 100 m

Men had higher odds of mobility limitations with higher level of physical workload and a exposure–response relationship was observed (Fig. 1). Introduction of mediators and confounders attenuated the results; however, men with high exposure had higher odds of limitations running 100 m also after the adjustments (Models 2 and 3). Among women, there was a tendency towards an association between higher level of physical workload and higher odds of limitations in running 100 m. However, the associations were not statistically significant for the least exposed group. Furthermore, women with high exposure had lower odds of limitations compared to women with moderate exposure.

### Limitations in climbing stairs

Men had a strong exposure–response relationship between level of physical workload and odds of limitations climbing stairs in unadjusted analyses. Including age increased the associations slightly. As seen in the two previous analyses, introduction of confounders and mediators attenuated the associations, but the associations were still statistically significant, when lifestyle factors were included in model 3, except in the lowest exposure-group. Among women, the results were comparable to the other outcomes, and there was no statistically significant association between exposure and limitations in climbing stairs after adjustments in Model 2 and 3.

The outcome measures are associated, and Pearson correlation coefficients have been calculated: walking and running: men (*n* = 4996): 0.399 (*p* < 0.0001) and women (*n* = 2247): 0.395 (*p* < 0.0001). Walking and climbing stairs: men (*n* = 4996): 0.513 (*p* < 0.0001) and women (*n* = 2247): 0.506 (*p* < 0.0001). Running and climbing stairs: men (*n* = 4996): 0.637 (*p* < 0.0001) and women (*n* = 2247): 0.681 (*p* < 0.0001). The statistical association between self-reports and objective measures of physical performance was high, calculated between performance in chair-rise test and mobility limitations (gamma coefficient 0.58 for men and 0.63 for women).

### Attrition analysis

Attrition analysis with data from Danish registers (Avlund et al. 2014; Lund et al. 2015) found that respondents to the CAMB questionnaire had higher educational level, and fewer respondents were un-employed, compared to non-respondents (12% vs 25%). Respondents had fewer contacts with their general practitioner during 2009 compared to non-respondents, and non-respondents had higher mortality between 2009 and 2012.

## Discussion

This study aimed to assess the association between cumulative physical workload, measured as lifting in working life and physical function in midlife, using self-reported mobility limitations as outcome. We found a statistically significant association between cumulative physical workload and increased risk of mobility limitations in men and women. Our results are in line with previous studies in this field suggesting increased risk of mobility limitations in participants with high physical workload (Leino-Arjas et al. 2004; Mänty et al. 2014; Hinrichs et al. 2014). However, the previous studies focused on the variation in effect on mobility, comparing the effect of occupational workload and leisure-time physical activity. We included leisure-time physical activity as a confounder or mediator in our analyses and found that the associations were attenuated. Inclusion of confounders and mediators explained a large part of the observed increased risk of mobility limitations, especially among women.

In previous studies in this cohort, we found a slightly negative association between cumulative physical workload and chair-rise performance among men, but not among women (Møller et al. 2015). However, the differences were small, and exposures in working life explained only a minor part of the variation in physical performance. Compared to those findings, the associations in the present study are slightly stronger, suggesting a negative effect of cumulative physical workload on later mobility, especially among men, and we observed an exposure–response relationship. To analyse these results further, we have studied the association between self-reports and objective measures and found high gamma coefficients indicating a strong association. We have chosen three different outcome measures that are obviously associated also statistically. However, the outcome measures were chosen to cover variations in physical capacity from having problems with walking (the less demanding and therefore less prevalent outcome), to problems with running and walking the stairs. We found a high correlation between the three outcome measures, especially between running and walking the stairs, the most physically demanding tasks. The results are expected, given the age of the population, and it could be argued that problems with walking are not relevant in this population.

In previous studies in this cohort (Møller et al. 2013, 2015), the participation rate was lower (31.1% compared to 40% in this study), since the participants had to show up for the health examination. Therefore, this study was planned to include more participants in the analyses of associations between physical workload and physical performance. From attrition analyses, we know that educational level and labour market attachment varied among participants in the health examination and participants responding to questionnaire only (Avlund et al. 2014). Furthermore, participants in the health examination were exposed to fewer physical exposures at work compared to participants not showing up for physical tests (Møller et al. 2015). Therefore, this study population may not be representative of the background population. However, introducing relevant confounders in the statistical models, decreases the risk of selection bias.

In this study, and in our previous studies, we have observed gender differences in the associations between workload and physical performance (Møller et al. 2013, 2015). In all previous analyses, the association between workload and physical performance was stronger among men, and confounders and mediators attenuated the associations more among women. In contrast, Leino-Arjas et al. found that female gender increased the risk of poor physical function after 28 years of follow-up (Leino-Arjas et al. 2004). Hinrichs et al. found that vigorous occupational activity increased the risk of mobility limitations to an almost similar extent among men and women except from in their fully adjusted model, where the association among women were statistically insignificant (Hinrichs et al. 2014). Mänty et al. found no significant interaction in the effects of physical workload on mobility regarding gender, and men and women were analysed in combination (Mänty et al. 2014). Compared to these previous studies, we find gender differences in the size and the direction of the associations. Men with a high level of physical workload had the highest odds of mobility limitations, whereas women with a high level of physical workload had odds comparable to women with low and moderate levels of physical workload. This finding is in contrast to other studies in the CAMB cohort, where Hansen et al. found a sharp gradient in physical function according to social class among both men and women (Hansen et al. 2014a). However, social class is not equivalent to physical workload, and the association between social class and physical workload has not been studied, yet. On the other hand, in a study of the influence of psychosocial factors at work on the incidence of mobility limitation during 6 years of follow-up in the DALWUH cohort, Hansen et al. found results concerning gender differences comparable to ours. High work pace was strongly associated with higher risk of incident mobility limitations in men but with lower risk among women (Hansen et al. 2014b). Differences in cumulative workload could explain some of our findings. The unexposed group represents the largest group both among men and women. The group of women with highest level of physical workload is small (10% of women have more than 20 exposure-years compared to 20% of men), and the observed differences could be an example of a “healthy worker effect” among women. Holtermann et al. found a U-shaped relationship between occupational physical activity and all-cause mortality among women (compared to a more linear relationship among men) (Holtermann et al. 2012). This gender difference is to be investigated in the future; it may be explained by different tasks at work or different physiological responses to physical workload among women.

### Strengths and limitations

The measure of cumulative physical workload is a strength compared to previous studies in the field (Leino-Arjas et al. 2004; Mänty et al. 2014; Hinrichs et al. 2014), since physical workload can vary during working life. Another strength is the use of a validated job exposure matrix which eliminates the bias of self-reported occupational exposures. Studies have concluded that self-reports of mechanical exposures are prone to recall bias and are affected by current health state, mental state and work environment (Wiktorin et al. 1999; Koch et al. 2016; Pedersen et al. 2016; Urda et al. 2017). For example, people with existing health problems, such as back pain, tend to overrate the physical work exposures (Mikkelsen et al. 2007). A JEM can eliminate some of this bias, but, expert-based JEMs provide only a crude measure of occupational exposure and have limitations. The lower body JEM is based on homogeneous exposure groups (Rubak et al. 2014), but, within each group, there could be substantial differences in exposures, and this could lead to differential misclassification (Kauppinen et al. 1992; Mannetje and Kromhout 2003). Also, the expert ratings of exposure were performed in 2008, without taking into consideration the change in work environment in general, and in exposures specifically, during the last 30 years. It was previously shown that physical workload decreased among men between 1970 and 1993 but not among women (Torgén and Kilbom 2000). Furthermore, in a JEM, it is assumed that exposures vary in the exposure groups according to age, hypothesizing that younger employees have more physically strenuous job tasks compared to older employees in the same exposure group (Torgén and Kilbom 2000). A job exposure matrix does not assess these types of age-related differences in exposure. In general, use of JEMs has pitfalls, and an objectively measured exposure during working life would have been preferred.

Another strength of this study is the relatively large sample size, since the data collection now has been finalized, and the last cohort in CAMB (the CPC) has been included in the analysis. Including participants from CPC increased the sample size and especially the number of women compared to our previous studies in the CAMB cohort, though tests of the effect of cohort as a variable in the analyses had no effect on the associations (results not shown). A limitation in this study is the inclusion of all participants at baseline, also participants out of work. However, participants out of work in midlife could have had up to 20 or 25 years of work despite being retired, and therefore they were included in the analyses. To study this problem, we repeated the analyses with participants employed at baseline, and the associations were attenuated. However, the trends were similar indication higher odds for mobility limitations among men with increasing physical workload. The odds for mobility limitations among women were further attenuated.

The study design is also a limitation. To study the genuine exposure–response pattern, a cohort study following participants over time should be performed including objective measures of outcome.

In conclusion, exposure to physical workload throughout working life is associated with increased odds of mobility limitations in both men and women. However, the associations are attenuated when possible confounders are added in the statistical models.
